# Anaesthetic Management of 2 Micropreemies with Difficult Airway: Case Report and Review of Literature

**DOI:** 10.5152/TJAR.2021.21180

**Published:** 2022-06-01

**Authors:** Anju Gupta, Nishkarsh Gupta, Pooja Singh, Kiran Kumar Girdhar

**Affiliations:** 1 Department of Anaesthesiology, Pain Medicine and Critical care, AIIMS, New Delhi, India; 2 Department of Onco-Anaesthesiology and Palliative Medicine, DRBRAIRCH, AIIMS, Delhi, India; 3 Department of Anesthesiology, VMMC and Safdarjung Hospital, New Delhi, India

**Keywords:** Airway management, newborn, perioperative care, videolaryngosocpe

## Abstract

The infants are explicitly vulnerable to develop anaesthesia-related complications, with micropreemies being at the end of the spectrum because of their unique morphological and physiological features. Airway changes in pediatric patients are most exaggerated in these tiny infants and their immature lungs provide a little reserve, and therefore, securing airways can be challenging in this population. Moreover, many devices available for managing difficult airways in adults are not available for use in this miniature version. Videolaryngoscopes are a useful addition to anaesthesiologists’ armamentarium but for micropreemies, size of videolaryngoscope can be a limiting factor as no videolaryngoscope comes in “00” size. The technique of videolaryngoscope may need to be modified in these patients. Regional anaesthesia can be an invaluable component of their perioperative care to facilitate a smooth recovery. The successful management of these vulnerable neonates requires a multidisciplinary team approach to maintain perioperative homeostasis of their immature organ systems.

## Main Points

- Anaesthesia management in micropreemies is challenging.
- Videolaryngoscopy should be considered for initial intubation attempts in micropreemies.
- Videolaryngoscopy in these infants with big floppy epiglottis should include epiglottis within laryngoscopy for better glottic exposure.

## Introduction

Anaesthesia for micropreemie has ramifications much beyond that of a term neonate and is much more complex due to immaturity of almost all organ systems.^1,2^ Literature is deficient regarding the anaesthetic management of these patients. We are reporting the airway management of 2 micropreemies posted for emergency surgeries and a brief overview of the anaesthetic considerations for this group of patients. Informed consent was obtained from the parents of both children for publication of the case details and images. 

## Case Presentation

Case 1: A 1-day-old male baby weighing 850 g, born at 29 weeks gestation, was posted for tracheoesophageal fistula (TEF) repair. His chest X-ray showed pneumonitis on the left side with reduced air entry on auscultation. Operating room (OR) was prewarmed to 30°C and baby was covered with warmed gauze pads. After attaching standard monitors and injection of caffeine 10 mg IV, the baby was preoxygenated with 100% oxygen for 3 minutes. Anaesthesia was induced with injection of thiopentone 3 mg, fentanyl 1 µg, and succinylcholine 1.5 mg. After ensuring ventilation with a two-hand technique and use of size 00 oropharyngeal airway, intubation was attempted using a Miller 0 blade. Cormack Lehane (CL) grade 4 view was obtained, and the oxygen saturation decreased to 88%. McGrath MAC videolaryngoscope (VL) size 1 also resulted in a CL grade 4 view. A repeat attempt by a senior anaesthetist after defogging with warm saline resulted in a CL grade 3a with optimum external laryngeal manipulation (OELM) and head positioning, but intubation failed, and saturation decreased to 85%. Subsequent use of size 0 CMAC Miller blade (Karl Storz, Tuttlingen, Germany) revealed a CL grade 2a with OELM and intubation was successful with a styletted 2.5 mm ID endotracheal tube (ETT) within 25 seconds. Anaesthesia was maintained with isoflurane (0.8%-1%) in O_2_/air. During the procedure, baby desaturated to 77%-80% on a few occasions but promptly responded to the release of lung retraction and recruitment maneuvers. Hypotension was managed with titrated fluid boluses and low-dose dopamine infusion (5 μg kg^-1^ h^-1^). The intubated baby was shifted to neonatal intensive care unit (NICU) because of inadequate ventilation and died of respiratory failure on postoperative day 2.

**Figure 1.**
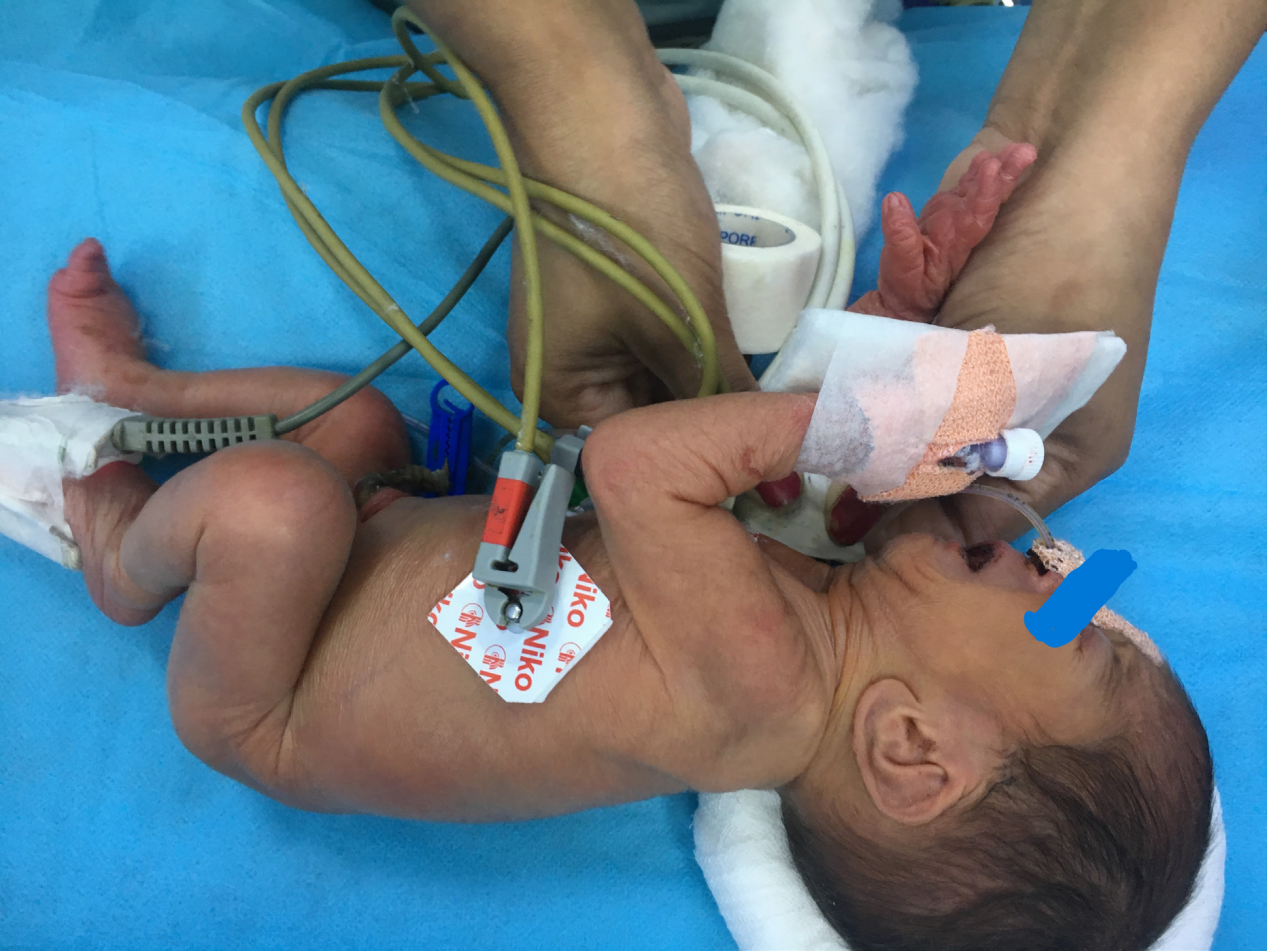
Micropreemie high anorectal malformation for colostomy.

Case 2: A 1-day-old female preemie, weighing 900 g with high anorectal malformation, was scheduled for colostomy (Figure 1). She was born at 28 weeks gestation out of normal vaginal delivery. The baby was transferred to prewarmed OR in a warmed isolette. After attaching standard monitoring, the heart rate (HR) went down to 90/min which improved to 130-140/min with injection of atropine 100 μg. The baby was preoxygenated with 100% oxygen for 3 minutes and premedicated with fentanyl 1 µg IV. Anaesthesia was induced with thiopentone 5 mg, and muscle relaxation was achieved with injection of atracurium 0.5 mg. An intubation attempt with Miller size 0 blade resulted in a CL 3a view, but the attempt was aborted as the baby desaturated. Second attempt with CMAC Miller 0 blade resulted in a CL 2b view with OELM, but intubation was difficult, and baby desaturated again. During the third attempt, anaesthesiologists lifted the epiglottis directly with a tip of CMAC Miller blade to obtain a CL grade 1 view and intubation within 18 seconds with 2 mm ID uncuffed ETT. After intubation, caffeine 8.5 mg was given IV, and anaesthesia was maintained on sevoflurane in oxygen/air. Intraoperative course was uneventful except one episode of bradycardia (HR suddenly dropped to 84/min) that responded to atropine 100 μg IV. The baby was extubated on the table and was shifted to NICU where the patient was sustaining well on nasal CPAP till 2 weeks of follow-up.

## Discussion

In view of the immature organ systems and altered anatomical features, micropreemies present a great challenge to the anaesthesiologist.^1,2^ Common conditions prevalent in these patients include broncho-pulmonary dysplasia, patent ductus arteriosus, transitional circulation, retinopathy of prematurity (ROP), altered temperature regulation, and apneic episodes. They are prone to develop alveolar collapse due to lack of surfactant and hypoxaemia.^1,3^ There were multiple episodes of hypoxaemia in the TEF preemie which were managed by removing lung retraction and giving recruitment manoeuver. Under anaesthesia, the cardiac output in micropreemies cannot increase rapidly because of high resting HR, immature ventricular muscles, low blood volume, and myocardial depression.^3^ Hypotension in the present cases was dealt with careful fluid bolus and titrated vasopressors. 

Airway-related differences in infants are much more exaggerated in micropreemies. Functional reserve capacity is low and oxygen consumption is high, so, their safe apnea time is very brief which further compounds intubation difficulty.^4^ Moreover, their airway dimensions are small, and mucosa is prone to edema following even mild trauma, hence multiple or traumatic attempts can compromise the airway. 

Videolaryngoscopes have camera placed near the tip of the blade, permitting anterior magnified view. Most trials on VLs in children have excluded preterm infants. A Cochrane metaanalysis concluded that there was insufficient evidence to recommend the use of VL for endotracheal intubation in neonates.^5^ The probable reasons could be the lack of requisite skill, lack of much-supporting evidence, lack of appropriate size VL, and safety issues. A relatively longer VL blade (size 0 or 1) when inserted conventionally with its tip positioned in the vallecula may push the epiglottis down over the glottic aperture as happened to us while using McGrath MAC VL size “1.” C-MAC^®^ Miller blade is designed based on conventional straight blade, has a shorter web as compared to Macintosh blade, and hence requires less mouth opening for ETT insertion and manipulation.^6^ So, changing over to CMAC Miller size “0” blade led to easy intubation with the aid of OELM and stylet. The Neonatal Resuscitation Program also recommends the size-0 Miller laryngoscope blade for premature neonates, but they do not specifically mention anything about micropreemies.^7^

Sinha et al^8^ documented good glottic views, 86.5% first attempt success rate, and intubation time of 22 seconds during intubation of 37 preterm and ex-preterm babies with C-MAC^®^ VL Miller blade size-0. However, their study findings should be cautiously extrapolated to extremely preterm babies as the smallest baby was 33 weeks post-gestational age and weighed 1100 g. They kept the tip of the blade in vallecula and found difficulty in directly lifting the epiglottis in few infants with long epiglottis. In our cases, lifting epiglottis indirectly could not expose the glottis and directly lifting epiglottis with CMAC Miller blade tip helped achieve successful intubation.

We had taken precautions to manage complications like hypothermia, hypoglycemia, and ROP in our cases.^9^ Preterm neonates are prone to postoperative apneic spells for 48-72 hours especially in anemic. We had given 10 mg kg^-1^ of caffeine prophylactically in both cases to reduce postoperative apnea.^10^

## Conclusions

The airway management of these tiny neonates can be very tricky due to narrow passages of upper airways and lack of cardiopulmonary reserves. Videolaryngoscope should be considered for initial intubation attempts in micropreemies and during VL one should include epiglottis within laryngoscopy for better glottic exposure.
